# Editorial: Cerebellar development and medulloblastoma: new players and therapeutic options

**DOI:** 10.3389/fcell.2023.1214635

**Published:** 2023-05-15

**Authors:** Manuela Ceccarelli

**Affiliations:** ^1^ Department of Hematology/Oncology and Cell and Gene Therapy, Bambino Gesù Children’s Hospital, IRCCS, Rome, Italy; ^2^ Institute of Biochemistry and Cell Biology, National Research Council (CNR), Rome, Italy

**Keywords:** cerebellum, medulloblastoma, spinocerebellar ataxia type 3 (SCA3), cerebellar mutism syndrome (CMS), autism spectrum disorder (ASD), cancer metabolism, cerebellar development

The cerebellum plays a pivotal role in the control and regulation of motor functions, contributing to the coordination, precision and timing of movements; in addition, recent evidence suggests its involvement also in cognitive and emotional functions, such as attention, memory, language and responses to fear or pleasure (Schmahmann, 2019). In humans, cerebellar development begins during early embryonic stages and persists for several months after birth. Granule cell precursors (GCPs) proliferate postnatally in the external granule layer (EGL), in response to Sonic Hedgehog (Shh) stimulus provided by underlying Purkinje cells; then, GCPs migrate inward to the molecular and internal granule layers (ML and IGL, respectively) and differentiate in mature granule neurons (Ruiz i Altaba et al., 2002). Abnormalities in cerebellar development are responsible for several disorders and cancer (Farioli-Vecchioli et al., 2012). Medulloblastoma (MB) is the most common malignant childhood brain tumor, arising in the posterior cranial fossa during early brain development. It is a heterogeneous disease, comprising four molecular variants (Wnt, Shh, Group 3, and Group 4) with different cells of origin, age of onset and prognosis. The current MB treatment includes maximal safe resection, chemotherapy and radiotherapy; however, survivors suffer devastating side effects, such as neurological cognitive and behavioral deficits (Juraschka and Taylor, 2019).

Thus, this Research Topic has focused on the discovery of signaling pathways or genetic mutations with significant impact on both the physiological and pathological cerebellar development, for the identification of new molecular targets/biomarkers of diseases and the generation of advanced preclinical models for the study of cerebellar pathologies.

Xiao et al. demonstrated that abnormal development of the cerebellum can lead to significant impairments in motor coordination and learning, deficits commonly observed in patients with autism spectrum disorders (ASD). Specifically, the authors observed that in a BTBR T^+^ Itpr3^tf^/J mouse model of autism, severe dystonia-like behavior and motor disfunctions were closely related with increased proliferation of GCPs in EGL and enhanced cerebellar foliation. Furthermore, in the BTBR cerebellum, Purkinje cells, the only efferent cerebellar neurons with a key role in motor function, showed morphological hypotrophy, with increased dendritic spine formation and suppressed maturation. Transcriptional analysis revealed a differential expression of genes of the TRPC family, encoding for non-selective Ca2^+^ channels, which regulate cerebellar neurogenesis and synaptic formation, suggesting their involvement in the pathological process of ASD.

Selective deficit of Purkinje cells’ function was also observed by Jansen-West et al. in a new mouse model of spinocerebellar ataxia type 3 (SCA3), obtained using adeno-associated virus technology. SCA3 is a dominantly inherited cerebellar ataxia caused by the expansion of a polyglutamine repeat in the protein ATXN3, which accumulates in neurons of patients resulting in neurodegeneration, progressive loss of locomotor control and eventual death. Jansen-West et al. demonstrated that in SCA3 mice the severity of locomotor deficits is closely linked with the extent of Purkinje neurons death and decrease of the ML thickness at the posterolateral fissure: this suggests a specific polyQ-ATXN3 toxicity in the cerebellar cells, probably due to changes in the expression of voltage-gated potassium channels leading to an increase in cell intrinsic excitability.

Cerebellar Mutism Syndrome (CMS) is a known and serious complication affecting approximately one quarter of pediatric patients undergoing surgical resection of posterior fossa tumors. In the acute phase, as early as 24 h after surgery, children with CMS show language impairments up to mutism, and emotional lability; sometimes, hypotonia and cerebellar motor deficits, brainstem dysfunction and cranial neuropathies may be associated. Usually within 6 months, children recover from CMS, but continue to have residual motor, behavioral, and cognitive deficits. Fabozzi et al. in their review aim to present the most recent knowledge on CMS, focusing above all on the management and rehabilitation of post-operative speech impairment. Notably, Fabozzi et al. underline how MBs belonging to the Wnt, Group 3 and Group 4 molecular subgroups are a known risk factor for post-operative CMS because these tumors have an intimate relationship with the cerebellar structures, unlike of Shh-type MBs that develop within the cerebral hemispheres.

Two cell populations give rise to Shh MB: GCPs within the EGL and cerebellar neural stem cells (NSCs) residing in the subventricular zone. In both cell systems that mimic the early postnatal cerebellum *in vitro*, Abballe et al. demonstrated the crucial role of the β-arrestin1-E2F1-ac axis in the control of apoptosis and cell cycle exit. β-arrestin1 is a transducer for G protein-coupled receptors that in GCPs, in response to Shh stimulus, moves to nucleus where interacts with CREB and P300 on target gene promoters and enhances the acetylation of E2F1 (E2F1-ac). As a result, the transcriptional activation of p27 gene, involved in cell cycle arrest, and of pro-apoptotic target genes of E2F1-ac (Trp73, Caspase3, and Caspase7) occurs. Also in NSCs, where β-arrestin1 gene is epigenetically silenced to maintain stem cell features, the ectopic expression of β-arrestin1 enhances the transcription of cell cycle inhibitor p27 and pro-apoptotic E2F1-ac target genes.

Finally, Marabitti et al. investigated the metabolic alterations at the cellular and molecular levels that support the growth, survival, invasion, metastasis, and resistance to therapy of medulloblastomas. In this review, the authors summarize the intrinsic and extrinsic factors affecting metabolic changes in MBs, integrating them with heterogeneity both between tumor subtypes and within tumors. Notably, Marabitti et al. emphasize the translational potential of this aspect, pointing to currently available drugs that target MB energy metabolism as innovative treatment strategies to suppress MB progression and minimize relapse and post-treatment resistance, thereby improving the outcome of MB patients and the quality of life of survivors.

Collectively, these articles highlight that cerebellar development requires a correct and timely balance between cell proliferation, differentiation, migration and survival, and that a deeper characterization of these processes and the molecular players involved can contribute to the understanding of many different pathological conditions. In the era of precision medicine, this Research Topic focuses on the identification of the genetic, transcriptomic and metabolic characteristics of some of the major cerebellar diseases, and encourages the scientific community to continue research in this field for the development and application of new therapeutic treatments.
